# Nanoscale hyperthermia mesostructures for sustainable antimicrobial design

**DOI:** 10.1016/j.xcrp.2024.102081

**Published:** 2024-07-08

**Authors:** Ying Cui, Huan Wu, Shilei Zhang, Zhihan Zhang, Genhong Cheng, Ren Sun, Yuan Shi, Yongjie Hu

**Affiliations:** 1 Department of Mechanical and Aerospace Engineering, School of Engineering and Applied Science, University of California, Los Angeles, Los Angeles, CA 90095, USA; 2 Department of Microbiology, Immunology and Molecular Genetics, David Geffen School of Medicine, University of California, Los Angeles, Los Angeles, CA 90095, USA; 3 Department of Molecular and Medical Pharmacology, David Geffen School of Medicine, University of California, Los Angeles, Los Angeles, CA 90095, USA; 4 Lead contact

## Abstract

Sustainability is critical in addressing global challenges posed by prolonged pandemics that impact health, economies, and the environment. Here, we introduce a molecular engineering approach for thermoregulated antimicrobial management inspired by firewalking rituals. The study uses *in situ* spectroscopy and multi-scale modeling to validate a hierarchical design. Efficient light-to-thermal energy conversion is achieved by engineering the molecular band structure. Rapid nanoscale hyperthermia is facilitated through thermal engineering. This approach significantly reduces the half-life of pathogens such as *Escherichia coli*, influenza A, and severe acute respiratory syndrome coronavirus 2 (SARS-CoV-2) to 1.4 min while maintaining a low perceived temperature on human skin. Standard disease infection and epidemic models show this technology’s potential to flatten outbreak curves and delay peak infection rates, which is crucial during the early stages of pandemics when developing vaccines and antiviral drugs takes time. The scalable manufacturing and broad antimicrobial applicability hold great promise for controlling emerging infectious diseases and diverse bioprotective applications.

## Graphical Abstract

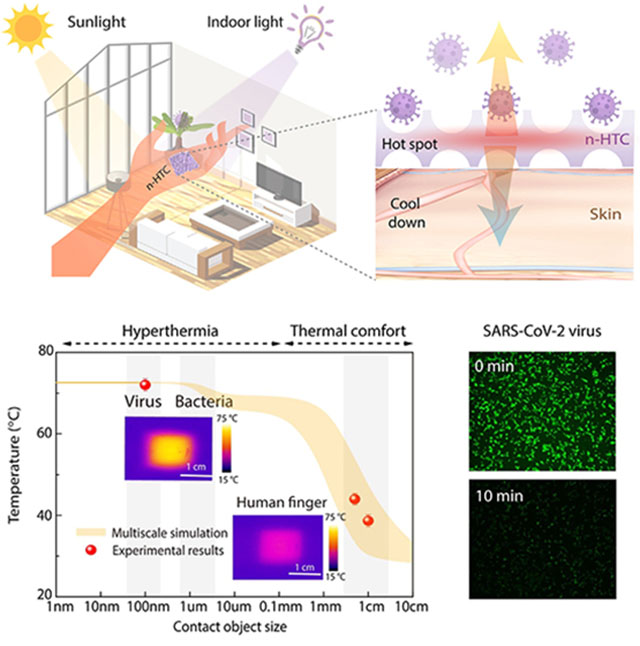

Cui et al. design a sustainable approach based on molecular and thermal engineering for antimicrobial technology, which can effectively suppress a broad range of pathogens under varied environments. Standard disease infection and epidemic models show that if adopted institutionally, this approach can help control future pandemic outbreaks.

## INTRODUCTION

**Figure 1.**
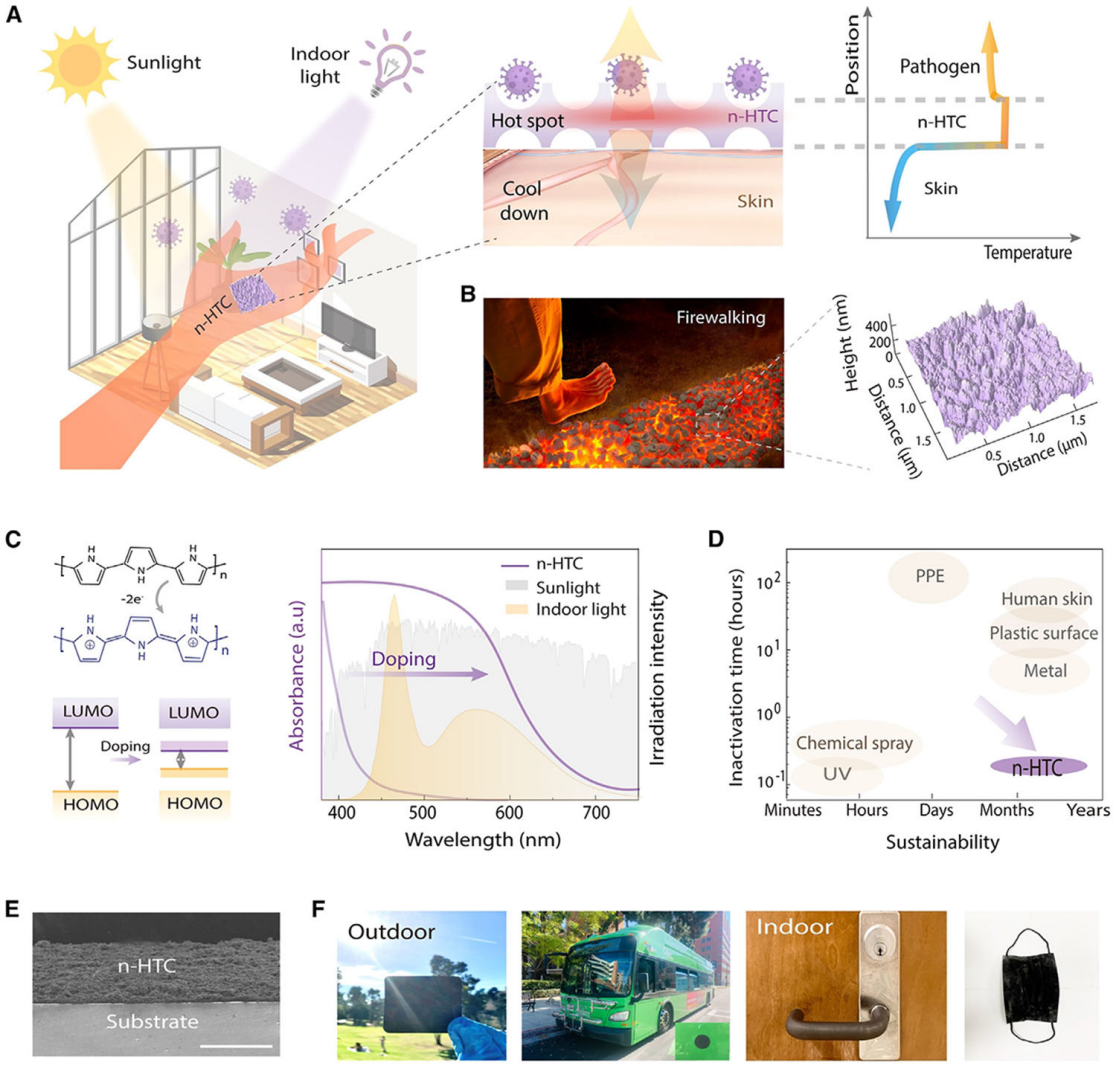
Materials design and processing for hierarchical thermoregulation comfort (A) Design concept to achieve nanoscale hyperthermia yet macroscale thermal comfort via hierarchical thermoregulation. At the nanoscale, sustainable light-thermal energy conversion and intact contact with pathogens to form local hot spot temperature for antimicrobial functions. At the macroscale, human interaction dissipates thermal energy and maintains a low perception temperature. (B) Firewalking-inspired design and the structural morphologies characterized by atomic force microscopy. (C) Chemical and band structures of the tunable photothermal molecules. UV-visible-near-infrared (IR) absorption spectra for the undoped and doped states, respectively, exhibiting enhanced light absorption of indoor and outdoor light, to facilitate efficient light-to-thermal energy conversion. (D) Illustration plot for the balance over the antimicrobial effectiveness and sustainability. (E) Cross-section scanning electron microscopy (SEM) images of n-HTC structures. (F) Sample adaptions for various objects under both indoor and outdoor environments.

Innovation in smart materials plays a crucial role in safeguarding global health amid ongoing public health pandemics caused by emerging infectious diseases. Exemplified by COVID-19, severe acute respiratory syndrome (SARS), avian influenza, and Ebola virus disease, high infection rates and significant burdens have been placed on healthcare systems.^1^ To combat these challenges, innovative materials designs preventing transmission and enhancing bioprotection have become essential.^2-10^ Studies on a range of public health interventions, including medical isolation, quarantine, contact tracing, social distancing, travel restrictions, and vaccination programs, have been widely adopted. However, the enduring stability of pathogens,^2-5,11-27^ which can maintain infectivity for hours in aerosols, up to 3 days on common surfaces,^28^ and even up to 7 days on heavily exposed personal protective equipment (PPE) like surgical masks,^29-31^ presents a persistent challenge. Fundamentally, developing advanced materials structures to effectively control viral persistence is critical in reducing infectious transmission.^1,17-24,32-50^ Intensive progress has been made in achieving effective disinfection.^17-24,32,35-51^ However, there remains a critical balance to be struck between effectiveness and sustainability, prompting extensive research (Figure 1D). Traditional disinfection techniques, like chemical and ultraviolet (UV) sterilization, often require daily or case-dependent application to maintain their intermittent microbial-fighting efficacy. This need places economic, environmental, and health burdens on both individuals and society. Maintaining broad and enduring antiviral conditions in an environmentally friendly manner continues to be a major hurdle. Additionally, the emergence of new viral strains and diseases may render existing protections and vaccinations inadequate, increasing infection risks for health workers and people in resource-limited settings. Further, indoor environments that are lit 24 h a day and highly foot trafficked, including hospitals, stores, airports, offices, and factories, are particularly prone to high levels of viral contamination.^17-23^ Consequently, there is an urgent demand for the development of bioprotective solutions that can be deployed universally, without geographical limitations, and offer simultaneous effectiveness and sustainability.^16-24,35,52,53^

In this study, we have developed a self-sustainable indoor and outdoor bioprotective structure via band-gap engineering and nanostructure thermoregulation. This design achieves high efficiency and continuous antimicrobial performance through optimized light absorption and nanoscale hyperthermia while maintaining wearable thermal comfort (n-HTC) during human interaction. At the molecular level, the optical absorbance was optimized to match the emission of light sources through adjustment of their molecular structures. At the nano- and microscales, we employed sustainable light-to-thermal energy conversion and a mesoporous thermal insulation layer. This hierarchical structure concentrates thermal energy and minimizes contact thermal resistance, creating a surface heat accumulation region for efficient antimicrobial heat transfer. On the macroscale, interaction with humans dissipates thermal energy, ensuring a low perception temperature and thermal comfort. Our scalable manufacturing process enables the application of n-HTC coatings to a wide range of objects and flexible structures in various environments. To assess its effectiveness, n-HTC was tested against multiple pathogens, demonstrating over 99.999% bactericidal and virucidal activity. Notably, it significantly reduced the persistence of SARS coronavirus 2 (SARS-CoV-2) on surfaces from several days to approximately 20 min (or a half-lifetime of 1.4 min). Additionally, our quantitative analysis of its antiviral effects suggests it could effectively suppress emerging infectious diseases. This self-sustaining, energy-efficient antimicrobial solution is adaptable to both indoor and outdoor settings, including hospitals, stores, airports, offices, and factories, where artificial lighting is a prevalent energy source.

## RESULTS AND DISCUSSION

### Hierarchical-thermoregulated comfort inspired by firewalking

Our design strategy of n-HTC was inspired by the ancient ritual of firewalking,^54-57^ which is the act of walking barefoot over a bed of hot embers or stones without burning, resulting from the low thermal conductivity of the stones and the rapid heat dissipation capabilities of the human body. To achieve hyperthermia at the nanoscale level while keeping wearable thermal comfort at the macroscale, this system integrates band-gap engineering and nanostructure thermoregulation mechanisms, as illustrated in Figures 1A and 1B.

In our molecular-level design (as shown in Figure 1C), we utilized a photothermal polymer based on doped-polypyrrole (PPy) to achieve efficient light-to-thermal energy conversion. This process is facilitated by the polymer’s ability to absorb a broad spectrum of light, particularly under illumination. The conversion mechanism is grounded in the excitation of loosely held electrons from the π to π* orbitals. These excited electrons then relax from a higher energy state (lowest unoccupied molecular orbital) back to the ground state (highest occupied molecular orbital), releasing heat in the process.

To optimize indoor light absorption, we adjusted the optical absorbance of the absorber to align with the emission spectrum of artificial light-emitting diode light sources. This was achieved by using citric acid as a dopant to reduce PPy’s band gap, thus enhancing its ability to absorb indoor light. In its undoped state, PPy acts as a thermal insulator but also an electronic conductor with a large band gap, absorbing only a small portion of light. However, doping alters the electronic property significantly. When one π-electron is removed from the polymer chain during doping, two localized electronic levels appear within the band gap. Further oxidation, which involves removing a second electron, leads to the formation of a bipolaron and the emergence of narrow bipolaronic bands.^58,59^ This change effectively decreases the energy gap and expands the spectrum of indoor light absorption in the 380–740 nm range. The UV absorption spectra for both undoped and doped states, as illustrated in Figure 1C, demonstrate how, through molecular structure adjustments and band-gap optimization, efficient indoor light absorption is achieved, facilitating enhanced light-to-heat conversion.

At the nanoscale, our development focuses on a mesoporous nanostructure thermoregulation system that employs nanoscale hyperthermia. This system is designed to interact effectively with both microscopic pathogens and macroscopic human beings. We utilize a spray-on interfacial polymerization approach^46,47^ (supplemental information) for the uniform coating of light-absorber nanoparticles on various mediums. This process involves creating a colloidal suspension of nanoparticles by mixing monomer pyrrole, ammonium persulfate oxidant, and dopants, which is then dissolved in liquid solvents to achieve a controlled evaporation rate and uniform deposition according to surface morphology. The suspension is sprayed onto substrates to initiate oxidative polymerization, resulting in thin-film coatings after natural evaporation. Due to elastic instabilities during surface polymerization over large areas, the resulting n-HTC structure exhibits a randomly distributed mesoporous morphology with feature sizes of approximately 200 nm, as confirmed by atomic force microscopy (Figure 1B, right). The spray-coated mesoporous n-HTC structure offers thermal insulation with low thermal conductivity, enabling surface heat accumulation. The interaction between the mesoporous n-HTC and nano- to microsized pathogens is efficient due to minimal contact thermal resistance, facilitating effective heat transfer for hyperthermia. At the macroscopic level, the larger contact thermal resistance hinders heat transfer from n-HTC to humans, mainly because of the air gap between the n-HTC and the human skin. Combined with the higher thermal capacity of human skin, this results in a low perception temperature, providing thermal comfort (Figure 1A).

At a larger macroscale, the n-HTC has been adapted to various structures and surfaces with high flexibility, such as plastics, medical masks, doorknobs, and public transportation vehicles, which are heavily exposed and potentially have high transmission rates in practical settings (Figures 1E and 1F). The wide applicability of the process allows the n-HTC to serve as a potential intervention against multiple modes of protection, modulating contaminated surfaces, objects, respiratory droplets, healthcare settings, and PPE in both outdoor (e.g., public buses, parks) and indoor (e.g., hospitals, stores, airports, offices, factories) environments for sustained effectiveness and long-term stability.

### Thermal transport measurements and modeling

**Figure 2.**
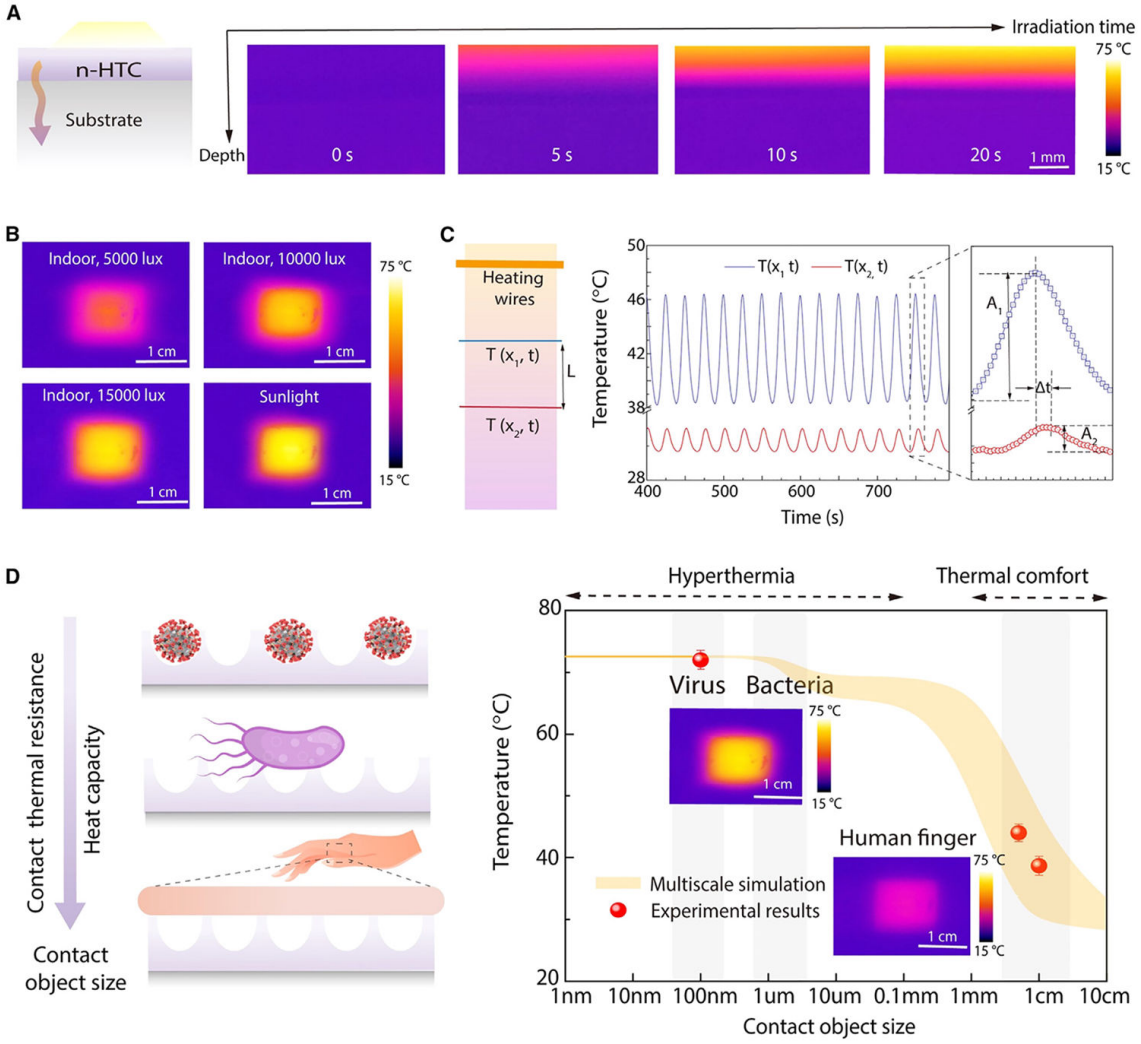
Thermoregulation principles by nanoscale heat accumulation and contact object-dependent temperature profiles (A) Irradiation time-dependent heat accumulation recorded by IR images (B) Steady-state temperature rise with different indoor and outdoor light radiation recorded by IR images. (C) Thermal conductivity measurements of n-HTC using the Angstrom method. (D) Experimental measurements (symbol) and modeling prediction (shadowed background) for size-dependent contact temperatures, including the contact objects ranging from virus (~200 nm) and bacteria (~1 mm) to human finger (~1 cm). Inset at the bottom, IR images indicating the experimentally measured temperatures of n-HTC surfaces, respectively, in contact with objects with varied sizes: small pathogens (left) and transparent glass (right).

The hierarchical design of n-HTC enables nanoscale hyperthermia for antimicrobial functions while maintaining macroscale thermal comfort for human interaction. To understand and verify the temperature regulation mechanisms of n-HTC, we carefully examined the self-sustained hyperthermia and thermal properties by studying its cross-section and transient dynamics, as well as its interactions with objects of different sizes (including viruses, bacteria, and human fingers). Infrared imaging was used to record the transient temperature profiles of the n-HTC across its cross-section, from the top surface to the substrate, as depicted in Figures 2A and 2B. When exposed to a virus, the n-HTC rapidly heated up under both outdoor and indoor lighting conditions, achieving an equilibrium temperature of approximately 72°C (indicated in yellow) within about 30 s. Conversely, areas a few millimeters below the top surface maintained temperatures close to room temperature (indicated in purple). To quantify these observations, we recorded the time-dependent temperatures at various depths and present these results in the supplemental information (Figure S2).

In addition, to verify that the n-HTC structure has thermal insulation to enable surface heat accumulation, we performed a direct measurement of the thermal conductivity of n-HTC using the Angstrom method (Figure 2C).^60-62^ We fabricated a microheater and two temperature sensors using platinum wires. The two sensors, positioned at ×1 and ×2, were set parallel to each other, spaced a distance, L, apart. They provided direct measurements of the local temperatures when a sinusoidal heat pulse was applied through Joule heating by the microheater wire. The measured temperature responses (Figure 2C) displayed periodic oscillations in sync with the heat pulses but with a phase shift (dt) dependent on the distance. The thermal diffusivity (α) was determined using the temperature amplitude ratio and phase shift, following the formula $\alpha =\frac{L^{2}}{2\times dt\times \text{ln}\frac{A_{1}}{A_{2}}}$, where $A_{1}$ and $A_{2}$ are the temperature amplitudes measured at the two locations (Figure 2C). The n-HTC exhibited an ultra-low thermal conductivity of 0.049 W/m·K, which is substaintially lower than that of the intrinsic PPy polymer, which we attribute to interface thermal engineering^63-65^ of the mesoporous structures and interfaces.^48^ These measurements confirm that the n-HTC design, leveraging mesoporous molecular structures for thermal insulation and efficient light-to-heat energy conversion, effectively creates surface hot spots while limiting heat dissipation on a macroscopic scale.

The hierarchical concept has been further validated through both modeling and experimental characterization of the temperature responses to contact objects of varying sizes. First, the n-HTC film’s temperature, as depicted in Figures 2D, is quantified with the contact thermal model.^60,66,67^ In the thermal model, all factors are considered as the practical settings, including the daily light absorption, light-to-heat conversion, and multi-scale thermal transport under the interaction with objects ranging in size from 1 cm to 1 nm. For smaller entities like typical pathogens, viruses (approximately 200 nm), and bacteria (around 2 μm), the n-HTC film attains a high equilibrium temperature. Conversely, in human interactions with larger objects such as a finger, due to the larger heat capacity and lower contact thermal resistance compared to the surface hyperthermia layer, the temperature remains near room temperature, ensuring thermal comfort.^61^

This results in non-linear, size-dependent contact temperatures, as indicated by the orange shadow in Figure 2D. Specifically, the initial temperature drop is due to increased contact thermal resistance between the n-HTC and larger objects, while the subsequent decrease arises from the larger heat capacity of these objects. In comparison, simulations of a surface without the hierarchical structure exhibit only a single temperature drop, illustrating the impact of contact thermal resistance on equilibrium temperature. Experimentally, temperatures have been measured using infrared thermal imaging (insets, Figure 2D) and microthermocouples. The experimental data (represented by red circles in Figure 2D) align closely with the model predictions. When in contact with viruses or bacteria, the temperature reaches about 72°C, providing effective hyperthermia that can inactivate common pathogens. In contrast, the temperature of the n-HTC film under human finger-sized contact is measured to be around 38°C, lying within the comfortable thermal range for humans and preventing skin injury. This surface hyperthermia for smaller pathogens acts as the antimicrobial mechanism, disrupting their microscopic structures synergistically, as further explored in subsequent discussions.

### Antimicrobial mechanisms and performance

**Figure 3.**
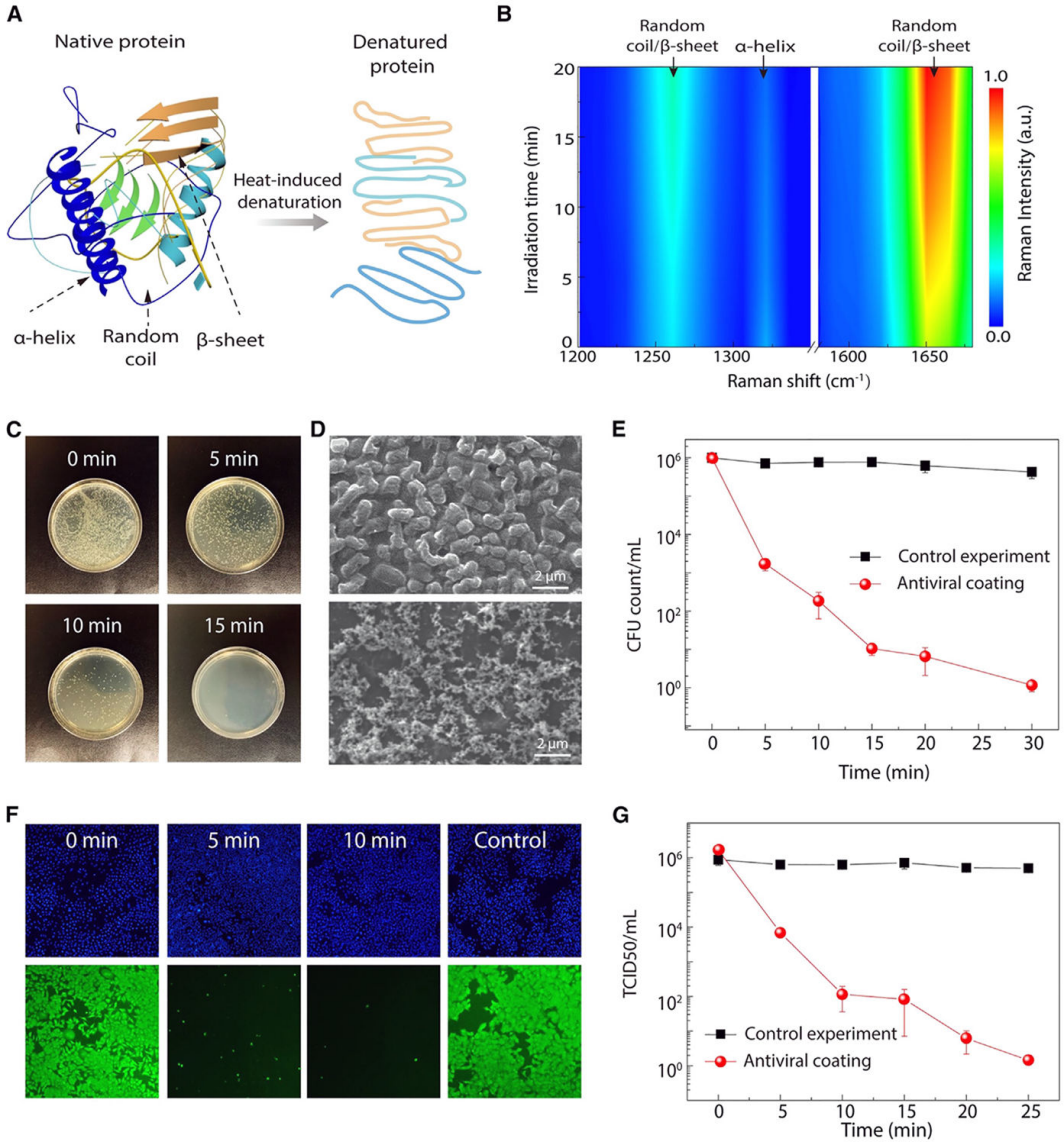
Temperature-induced inactivation mechanisms and antimicrobial performances (A) Temperature-induced protein denature illustration. (B) 2D Raman mapping of pathogens measured on n-HTC surface under different contact times. The Raman peaks indicate key protein secondary structures: α-helix, β-sheet, and random coil, as well as their time-dependent Raman intensity and structural transitions. (C) Agar plate photographs for *E. coli* under different contact times. (D) SEM images showing the *E. coli* morphologies in contact with n-HTC films under 0 (top) and 10 (bottom) min, respectively. (E) CFU counts of *E. coli* measured as a function of contact times. (F) Optical images (top) and fluorescence images (bottom) for human influenza virus under varied contact times. (G) TCID50 reading of influenza measured as a function of contact times. Error bars represent the standard deviation of three independent replicates.

To elucidate the temperature-induced inactivation mechanisms of pathogens, we employed Raman spectroscopy for *in situ* analysis of structural changes in proteins over time under the interaction with n-HTC. Time-dependent 2D Raman mapping (Figure 3B) and spectra (Figure S3) were obtained after varying the periods of contact/irradiation on the n-HTC surface (ranging from 0 to 20 min). These mappings and spectra correspond to characteristic secondary protein structures (Figure 3A), which are predominant structural elements in typical pathogens^68^:

1. The Raman band at 1,320–1,350 cm^−1^ is indicative of the α-helix structure. This involves amide groups stabilized by hydrogen bonds between the carbonyl (C═O) groups of each peptide bond parallel to the helix axis and the N─H group of the peptide bond in the amino acids below the helix.
2. The Raman bands at 1,220–1,280 and 1,610–1,680 cm^−1^ represent more relaxed structures, specifically β-sheets (stretches of polypeptide chains connected by backbone hydrogen bonds, forming a generally twisted pleated sheet) and random coils (randomly oriented monomer subunits without specific bonds, resulting in a statistical distribution of shapes).

Here, the α-helix is considered the most stable structure, representing the native “folded” state, while β-sheets and random coils are associated with denatured “unfolded” states.^69,70^ As the contact time on the n-HTC film increases (Figure 3B), Raman spectroscopy reveals a significant decrease in the intensity of α-helix bands, accompanied by an increase in β-sheet/random coil bands. Such intensity change results from a reduction in the fraction of α-helix structures and an increase in β-sheets and random coils within the proteins, signifying a structural transition of peptide units. Fundamentally, the temperature-induced transition can be understood due to the differences in configurational entropy and enthalpy.^71^

The observed evolution in Raman spectra confirms the thermodynamic transition from a native folded protein structure (α-helix) to a denatured unfolded structure (β-sheets and random coils) at elevated temperatures. This transition elucidates the protein denaturation process under hyperthermia, leading to disinfection. Importantly, this temperature-induced denaturation is a universal mechanism for antiviral disinfection, effective against various pathogens by disrupting their protein structures.

To evaluate such antimicrobial performance of the n-HTC, standard persistent lifetime tests were conducted by applying live pathogens on the n-HTC surface and measuring their infectivity decay over time. First, we examined the antibacterial efficacy of n-HTC using gram-negative *E. coli* as model bacteria. For the antibacterial assay, the n-HTC-coated surface was loaded with 1 × 10^6^ colony-forming units (CFUs) of a bacterial suspension, and its proliferation was assessed by agar plate counting. Viable colonies of *E. coli* after different exposure times were counted (Figure 3C) and shown to be sharply decreased after several minutes’ contact with n-HTC and diminished as quickly as after 15 min, indicating the highly effective antibacterial activity of the n-HTC coating. We also examined the morphological changes of bacteria: as shown in the scanning electron microscopy images (Figure 3D), pristine *E. coli* cells are of a smooth surface with rodlike structures. After contact with n-HTC, cellular deformation and surface collapse were found for these bacteria; most of the cells were lysed, and numerous small portions of debris were observed. Quantitatively, time-dependent CFU counts were recorded for *E. coli* in contact with both the n-HTC surface and a control surface (glass) under the same ambient conditions (Figure 3E). The control samples maintained the same level of CFUs during the whole incubation time. By contrast, a significant decrease of CFUs for *E. coli* on n-HTC was measured, with 4 and 6 log CFU reduction or effective contact-killing efficacies of 99.99% and 99.9999% within 10 and 30 min contact, respectively.

Next, we examined the antiviral efficacy of n-HTC on human influenza A virus. To test the viral persistent time, aliquots of influenza A virus were loaded on n-HTC-coated and -uncoated control substrates (glass and plastic) under the same ambient conditions. After each contact time, viral samples were retrieved from the substrate and quantified by measuring their infectivity in human lung epithelial cells (A549). At 16 h post-infection, cells were fixed and stained for influenza nucleocapsid protein (NP) (Figure 3F). NP staining (bottom row, Figure 3F) of the control samples at 30 min maintains a strong signal, indicating no loss of infectivity after prolonged contact with glass or plastic substrates. On the contrary, 5 and 10 min contact on the n-HTC-coated surface resulted in a significant loss of viral infectivity, as almost no NP signal can be detected. In addition, we quantified the viral titer by measuring the tissue-culture infectious dose (TCID50) per milliliter of each viral sample. Figure 3G shows a time-dependent influenza titer drop after contact with the n-HTC-coated (red) or control glass substrate (black). As early as 10 and 25 min after n-HTC-coated surface contact, the influenza virus has 4 and 6 log titer drops, respectively. In contrast, viral samples on control glass or plastic surfaces showed no apparent titer loss even after 25 min contact, consistent with our immunofluorescent result as well as literature reports on common surfaces.^27^

### Antiviral effectiveness against SARS-CoV-2

**Figure 4.**
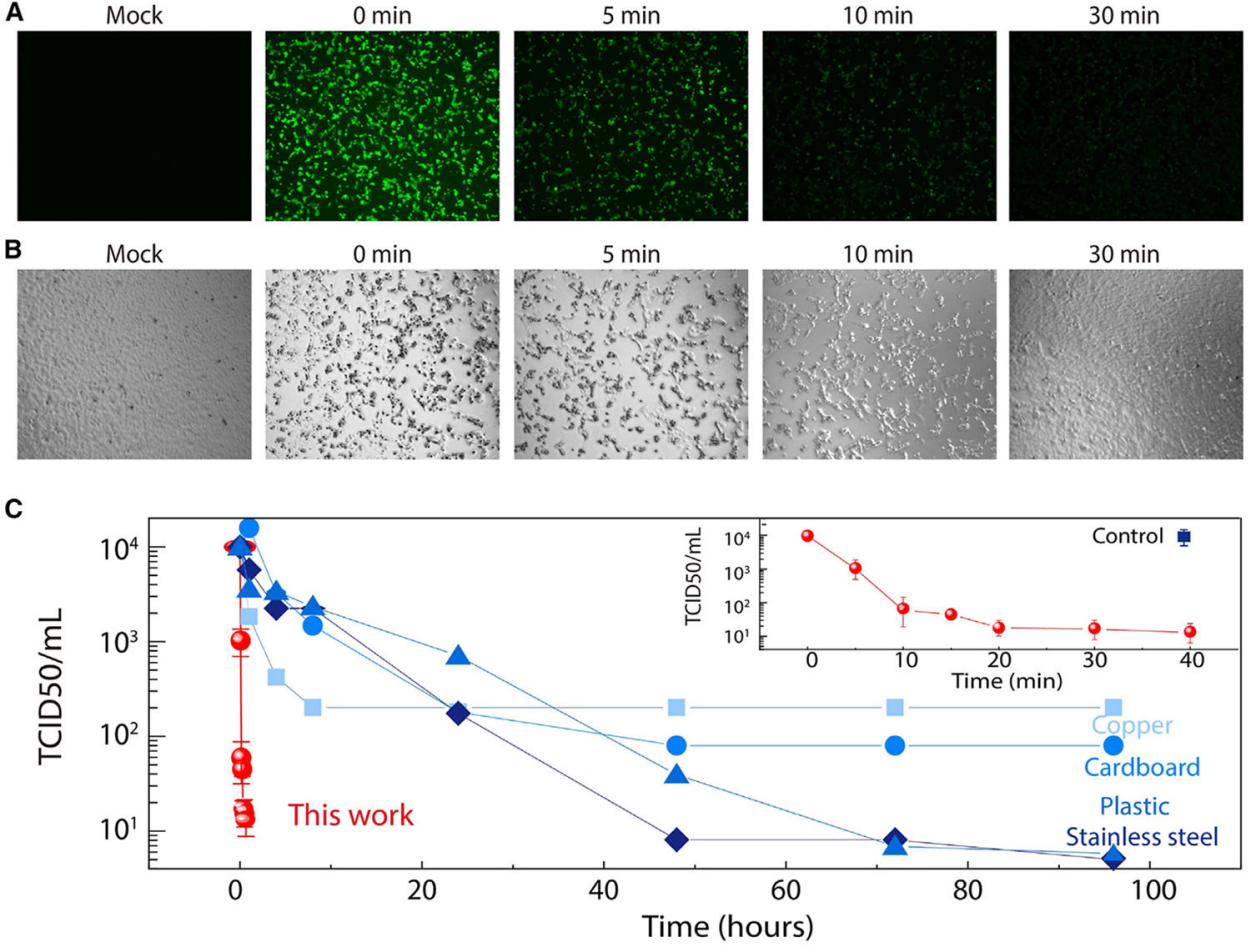
Experimental characterizations of viral persistence for SARS-CoV-2 (A and B) Fluorescent images (A) and bright-field images (B) for SARS-CoV-2 virus in contact with antiviral coating under varied contact times. Uninfected (mock) cells are included. (C) Experimental results of the SARS-CoV-2 titer response to contact times versus literature reports^28^ on varied objects. Error bars represent the standard deviation of three independent replicates.

As a further step, we investigate the antiviral effectiveness on live SARS-CoV-2 virus, the causative pathogen of COVID-19. SARS-CoV-2 has been reported to have a long persistent time under multiple conditions,^28-31,72^ which underlines the potential high-rate transmission routes during early cryptic SARS-CoV-2 dissemination. Here, live SARS-CoV-2 virus was applied on the n-HTC-coated surface for different periods of time, and the viral infection in Vero-E6 cells was measured. The green fluorescence images in Figure 4A indicate active infection mediated by live SARS-CoV-2 virus: as observed, as soon as after 5 min contact with the n-HTC surface, the viral titer of SARS-CoV-2 dropped significantly. After 30 min contact, there is barely any infectious viral particle left. Such a substantial antiviral effect is also observed in the bright-field images (Figure 4B), where a strong SARS-CoV-2-infection-mediated cytopathic effect of Vero-E6 cells can be observed. The degree of infectiousness on Vero-E6 cells exhibited a dramatic decrease as time increases, which demonstrates the highly effective inactivation of SARS-CoV-2 virus by the developed n-HTC. The data were quantified by a TCID50 experiment and are shown in Figure 4C: the viral titer of SARS-CoV-2 dropped from 1 × 10^4^ TCID50/mL to the detection limit within 20 min after contacting the n-HTC-coated surface. By contrast, the control surface shows no difference in terms of viral infectivity change for 40 min, which is the longest time we measured. Importantly, the data are plotted here for comparison in the context of other common surfaces^28^ (Figure 4C), where SARS-CoV-2 maintains its infectivity on multiple surfaces for a prolonged time. For example, it can stay infectious on copper for over 4 h, cardboard for 1–2 days, and stainless steels and plastics for 2–3 days. Dramatically, the n-HTC treatment on these surfaces enables efficient antiviral effects to disinfect SARS-CoV-2 from 3 days down to ~20 min. Quantified by the viral half-life time, the reduction is even significant, e.g., reduced from ~7 h on plastic to 1.4 min after n-HTC coating. From the comparison, these experimental studies clearly show that n-HTC coating is very effective in suppressing the persistent time of SARS-CoV-2, which highlights its application potential for emerging pandemic control.

### Sustainable bioprotective applications

**Figure 5.**
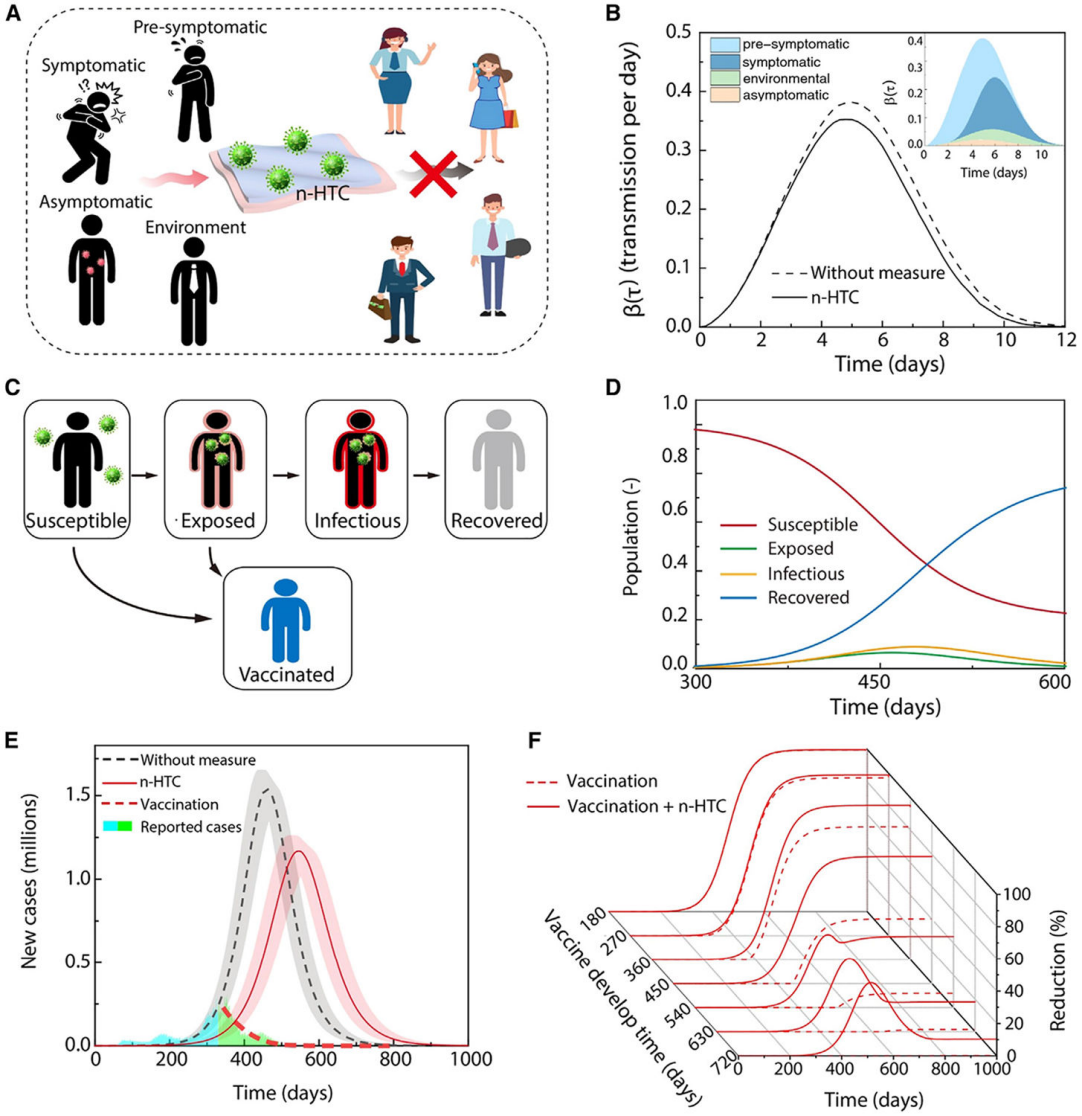
Modeling evaluations for emerging disease control and bioprotective applications (A) Schematic illustrating the transmission model of infectious diseases. The effect from n-HTC is determined by evaluating the transmission between known cases (black) and non-infectious people (colored). (B) The infectiousness function b(t) for standard persistent time without external measure^5^ (dashed line) and suppressed persistent time with n-HTC (solid line), respectively. The difference of the two curves indicates contribution from suppressed viral persistence time. Inset, the quantitative data and respective contributions to β(τ) from the four routes are adapted from Ferretti et al.^5^ (C) Schematic illustrating the epidemic model. (D) The susceptible, exposed, infectious, and recovered populations predicted by the SEIRV model. (E) The affected populations by COVID-19 as *a priori* predictions based on reported real cases^66^ before the US deployed vaccine (blue filled area) for future outbreak dynamics under varied conditions. The shadowed background represents modeling uncertainty. (F) The epidemic evolutions illustrating the combined effects from vaccination and n-HTC with the consideration of varied time lags required for vaccine development.

Lastly, we looked at the effects of antimicrobial effectiveness on emerging infectious disease control and sustainable bioprotective applications. Here, we adapt the recent analysis results^5^ to model the transmission of pathogens through four major routes (Figure 5A): symptomatic transmission (s),^3^ presymptomatic transmission (p),^73^ asymptomatic transmission (a),^74^ and environmental transmission (e).^3,75,76^ The infectiousness function can be decomposed into the same four contributions:

(Equation 1) $$\beta (\tau )=P_{s}\beta_{s}(\tau )+P_{p}\beta_{p}(\tau )+P_{a}\beta_{a}(\tau )+\int_{I=0}^{\tau }\beta_{e}(\tau -I)E(I)dI,$$

where $P_{i}$ is the proportion of respective individuals among all the infected populations and $\beta_{i}(\tau )\;(i=s,p,a,e)$ is the infectiousness function of the four transmission routes, respectively.^5,15^ E(I) is the rate of the contaminated surfaces infecting new individuals after a time lag I since being contaminated. As shown in Figure 5B (dashed line), the current β(τ) (without measure) was determined using standard persistent time for E(I) and has been reported in recent literature.^5^ To estimate the reduction of SARS-CoV-2 transmission contributed from viral persistence time reduction, we calculate the β(τ) using an updated persistent time of 20 min, the same as what we have measured on n-HTC. The reduced persistent time results in a reduction of the infectious function in Figure 5B (solid line). Quantitatively, the peak value of β(τ) is reduced from 0.38 to 0.35 transmissions per day. Correspondingly, the suppressed infectious persistent time from n-HTC leads to a responding reduction in $R_{0}$ (for example, ~10% in Figure 5B).^62,63^ Furthermore, to view the effects of the antimicrobial effectiveness for emerging infectious disease control, we applied the standard SEIRV model^77,78^ to evaluate the dynamics of infectious diseases in epidemiology. In particular, the timeline of a disease evolves through the interactions among five compartments (Figure 5C): the susceptible (S), exposed (E), infectious (I), recovered (R), and vaccinated (V) populations. In Figure 5D, the time-dependent population in each compartment is calculated, illustrating the dynamic evolutions of the pandemic. We used the reported cases of daily infection in US before the government’s deployment of a vaccine (blue filled area in Figure 5E)^79,80^ and fixed the total population at the US population to determine the modeling results under different conditions (i.e., without measure, n-HTC, vaccination) (Figure 5E), where the effect of viral persistent time is considered via the updated β(τ) (reduced from 0.38 to 0.35) from Figure 5B. In addition, we model the combined impact of vaccination and viral persistent time suppression such as n-HTC interventions on the variation of epidemic dynamics. The reduction in cumulative infected population, when comparing either vaccination only (dashed line) or vaccination on top of the viral persistent time suppression (solid line) with the worst-case scenario (without measures), are plotted in Figure 5F. We considered that a highly effective vaccine needs from 180 to 720 days after disease onset for development,^81^ a process that includes developing mRNA vaccine technology, going through clinical trials, FDA approvals, and administration to general populations. Our simulation shows that under such conditions, n-HTC application can further reduce the total infected population by up to 40%. This is especially important for future emerging pandemics because the development of vaccination takes time, and thus it is essential to mitigate the burden of bioprotective applications. In general, at the early stage of pandemics, prophylactic vaccines or pharmaceutical interventions such as antiviral therapeutics are not available.^81^ Therefore, such a sustainable and generic antipathogen intervention could become an important public health measure that individuals or communities can adopt to reduce infectious control and promote sustainable bioprotective applications.

### Summary and future perspective

In summary, we have developed universally applicable, highly energy-effective, and sustainable bioprotective structure through band-gap engineering and nanostructure thermoregulation. This structure achieves continuous hyperthermia for targeting microscopic pathogens while ensuring thermal comfort for human interaction, effectively bridging the gap between antimicrobial effectiveness and sustainability. The n-HTC structure demonstrates a significant reduction in the persistence of pathogens, as evidenced by experimental studies on both bacteria and viruses. Notably, the duration of SARS-CoV-2 viability is drastically reduced from several days to 20 min, with a half-lifetime of 1.4 min. Disease infection and epidemic model evaluations indicate that these antiviral structures could help to flatten the outbreak curve and delay the peak infection rate, potentially preventing healthcare system overloads, particularly in the early stages of pandemics when vaccine development is still underway.^81^ Overall, the n-HTC’s generic antimicrobial mechanism, which is based on localized hyperthermia, combined with its demonstrated scalable manufacturing process adaptable to various surfaces, positions it as an outstanding candidate for creating bioprotective equipment and building resilient health environments^82^ against future pandemics. We anticipate that such a sustainable design will help alleviate the economic, environmental, and health burdens faced by individuals and societies during prolonged pandemic challenges.

## EXPERIMENTAL PROCEDURES

### Resource availability

#### Lead contact

Further information and requests for resources should be directed to the lead contact, Yongjie Hu (yhu@seas.ucla.edu).

#### Materials availability

Materials generated in this study are available from the corresponding authors upon reasonable request.

#### Data and code availability

The authors declare that the main data supporting the findings of this study are contained within the paper. All other relevant data are available from the corresponding author upon reasonable request.

For further computational and experimental details, see the supplemental information. Figures S1-S4 show detailed results.

## Supplementary Material

1

## Highlights

Molecular thermal engineering for antimicrobial design and infectious disease control

Rapid nanoscale hyperthermia verified through experiments and multi-scale modeling

Suppress all pathogens while maintaining a low perceived temperature on human skin

Institutional technology adoption can break pathogen transmission and control pandemics
