# Supplementary material for: Nanoscale hyperthermia mesostructures for sustainable antimicrobial design
Source: Cell Rep Phys Sci. Author manuscript; Available in PMC 2024 Aug 1. (PMC11293369; doi:10.1016/j.xcrp.2024.102081)
Supplement: 1 [file NIHMS2010734-supplement-1.pdf]

Cell Reports Physical Science, Volume *5*

Supplemental information

Nanoscale hyperthermia mesostructures

for sustainable antimicrobial design

Ying Cui, Huan Wu, Shilei Zhang, Zhihan Zhang, Genhong Cheng, Ren Sun, Yuan Shi, and Yongjie Hu

Supplemental Experimental Procedures

Chemical preparations and materials characterizations. Analytical grade ammonium persulphate (APS) (Sd fine) was used as oxidizing agent. The chemical polymerization was carried out in a beaker by mixing 0.1 M aqueous solution of pyrrole and 0.2 M of APS and 0.05 M citric acid as dopant. The polymerization was carried out for a period of three hours. The product was washed successively by methanol followed by distilled water. The solution reaction is sustained with homogeneous dispersion, evaporation and viscosity of the solvent for uniform thin film deposition. Solution droplets are sprayed over application substrates to form solid thin films after natural evaporation. The surface and cross-section images are taken by a field-emission scanning electron microscope (SU-3500, Hitachi). Experiments were performed under 5000, 10000, 150000 illuminance (lux) LED light (white LED, 2700K) and solar simulator (Asahi Spectra) under 1 sun. UV absorbance studies were performed by UV/Vis/NIR Spectrometer (HP/Agilent 8453). Temperatures of the samples were measured by an infrared camera (FLIR A655sc) and a thermocouple (OMEGA). Temperature reading were made using the FLIR Tools+ (FLIR) and ImageJ (NIH) commercial software packages.

Thermal measurements. Thermal diffusivity is measured using the standard Angstrom method. A high resistance platinum wire was embedded in the sample and to apply a sinusoidal heat signal. Thermocouples applied to obtain the temperature profile the sample at different locations from the heater. Amplitude of temperature response decays along the length of the sample and simultaneously experiences a phase shift. Thermal diffusivity is obtained through the knowledge of temperature amplitude and phase shift through the equation, $\alpha = \frac{L^2}{2 \times dt \times \ln\frac{A_1}{A_2}}$, where L is the distance between temperature sensors, dt is the phase difference between two temperature responses, A_1 and A_2 are the temperature amplitudes measured at the two locations. A function generator supplies sinusoidal heat pulse to the heater. The sample is placed inside a high vacuum chamber to eliminate convective heat losses. Specific heat is measured using a differential scanning calorimeter (TA Instruments, 2920) with a temperature increase rate of 5 °C/min from room temperature to 100 °C. The thermal conductivity κ of n-HTC is obtained from specific heat Cp, density ρ, and thermal diffusivity α, using κ = αρ Cp.

Cells, bacteria, and virus cultures. A549 cells were cultured in RPMI-1640 with 10% FBS. VeroE6 cells were cultured in DMEM with 10% FBS. Both cells were from ATCC. K12 E. coli DH5α (Novagen) were grown in LB media. Influenza A/WSN/33 virus were generated using eight-plasmids reverse genetic platform. SARS-CoV-2 (USA- WA1/2020) was from UCLA BSL3 high-containment facility.

Antibacterial assay. 10 µl diluted E. coli bacterial suspension was spotted on the n-HTC samples surface for varied time (5, 10, 15, 20, 30, 40 mins) and then harvested by mixing with 1 ml of media, and the suspension was serially diluted ($\times 10^1$, $\times 10^2$, $\times 10^4$, and $\times 10^6$) to be plated on LB agar for the bacterial enumeration. All data were standardized as 1×10^6 CFU initial load and plotted CFU.

Antivirus assay. 10 µl diluted Influenza virus suspension was spotted on the n-HTC samples surface for a certain time (5, 10, 15, 20, 30, 40 mins) and then harvested by mixing with 1 ml of media, and the suspension was serially diluted ($\times 10^1$, $\times 10^2$, $\times 10^4$, and $\times 10^6$). Virus concentration was assessed by tissue-culture infectious dose [TCID50] measurement. 100 µl serial diluted influenza virus was used to infect A549 cells in complete media in six replicates. Infected cells were incubated at 37℃ with 5% CO_2 for two days. Cytopathic effects were examined for each infection and TCID50 were calculated using the Reed Muench method.

Immunofluorescent assay of influenza infected cells. Influenza infected A549 cells were incubated at 37°C with 5% CO_2. After 16 hours, cells were fixed with cold methanol, permeabilized with 0.1% Triton-X100, and blocked with 3% BSA and 10% FBS. Anti-NP monoclonal antibody (GeneTex) were used to detect influenza nucleocapsid (NP). DAPI was used for staining nucleus DNA.

SARS-CoV-2 experiments. SARS-CoV-2 live culture studies were performed under biosafety level (BSL-3) high-containment facility at UCLA. 10 µl SARS-CoV-2 GFP reporter virus at 1×10^4 PFU was used following the same literature conditions[48], and then spotted on n-HTC sample surfaces with varied contact time (5, 10, 15, 20, 30, 40 mins). At the indicated time point, the SARS-CoV-2 samples were suspended with 100 µl of PBS. The viral titers of SARS-CoV-2

suspension were measured by TCID50 assay in Vero-E6 cells. Briefly, the viral suspension was subjected to 10-fold serial dilution, and 100 μl of diluted viral inoculum was added to cells in eight replicates. After 3–4 days postinfection, the wells were examined for the observable CPE (cytopathic effect), compared to the mock-infected control. For fluorescence analysis of GFP expression in Vero-E6 cells infected with SARS-CoV-2 GFP reporter virus, cells were fixed for 20 min in 4% formaldehyde in PBS and washed in PBS. Fluorescence was observed using Leica Fluorescence Microscope, and images were captured and processed using LAS X Multi-channel acquisition software.

Multi-scale thermal modeling, contact temperature simulations and measurements. Size-dependent temperature profiles are determined by solving heat equations using finite element method. Multi-layer structures were considered, including antiviral coating layer (200 μm) and an application substrate (2 mm). Light absorption is considered with 1 sun and 1 μm penetration depth. The mesoporous structures and surface morphology of n-HTC is taken into account from the atomic force microscope and scanning electron microscope characterizations to effectively consider the enhanced thermal insulation and surface temperature accumulation. Standard boundary conditions are applied, including convection heat transfer on top surface and fixed temperature at the bottom surface. The size of contact objects is considered in three dimensions and the perception temperature is treated effectively as the volume-average of the contact objects and the thin hyperthermia layer (< 1 μm) on the top surface of n-HTC film. Therefore, the perception temperature, due to the very small thermal capacitance of the thin hyperthermia layer and the porous structure, as well as its very low thermal conductivity, strongly depends on the contact object sizes: The perception temperature is hyperthermia (~72℃) when contact object is small (e.g., pathogens), but is close to room temperature (~38℃) when contact object is large (e.g., human finger). The modeling results are compared with experimental measurements in Figure 2a, showing good agreement. For experimental verification, the sample temperatures were measured under contact objects with different sizes and cross-validated by two methods: thermal imaging using an infrared camera (FLIR A655sc) and temperature reading using a micro-size thermocouple (OMEGA).

Transmission model. We applied the standard SEIRV model to evaluate the epidemics. Such compartmental models are the most common approaches in epidemiology to predict the dynamics of infectious diseases. In particular, the timeline of a disease evolves through the interactions among five compartments (Figure 5c): the susceptible (S), exposed (E), infectious (I), recovered (R), and vaccinated (V) populations. The susceptible is the population who are at risk of becoming infected but have no contacts with the disease so far; the exposed is the population who have contacts with the disease but not get infected yet while they have a chance to become the infectious; the infectious returns to the recovered after recovering from the disease; the susceptible and the exposed become the vaccinated once they get immunized. Quantitatively, the epidemic dynamics is governed by the following differential equations,

$$\begin{cases} \dot{S} = -\varphi SI - \nu_1 S \\ \dot{E} = +\varphi SI - \alpha E - \nu_2 E \\ \dot{I} = +\alpha E - \gamma I \\ \dot{R} = +\gamma I \end{cases} \tag{2}$$

where φ, α, and γ are the transition rate coefficients among four compartments following literature values[5], and ν_1 and ν_2 are vaccination rates, i.e. the portion of the population get vaccinated per day, for the susceptible and the exposed, respectively, which were taken from the U.S. daily vaccination data and reported cases for COVID-19.

While the SEIR model is commonly used to predict pandemic dynamics, incorporating additional factors could enhance its effectiveness in modeling disease prevention and epidemic control. For instance, assessing reduced transmission due to shorter virus persistence times primarily attributes changes in the reproduction number, R0, to environmental factors. However, the distinction between different transmission routes could be refined[54], influenced by n-HTC specifications here. Transmission among individuals in prolonged close contact, for example, may not be attributed solely to environmental routes. The absence of n-HTC could decrease exposure and infection risks, as infectious doses often originate from more direct routes like exhaled droplets, which can be neutralized by n-HTC in confined spaces. By considering these aspects, the reproduction number could be further affected via other routes, though quantifying these reductions is challenging. Moreover, factors such as birth, infection-related mortality, general mortality for all compartments, physical quarantine, temporary immunity, and recovery enhance

the complexity of outbreak predictions and affect population distribution. The use of sustainable bioprotective measures could also lower transition rate coefficients between different compartments, not just from susceptible to exposed as seen in simplified models. This could lead to fewer peak infections, delay outbreak onset, and extend the inter-epidemic period. In general, public health systems might experience reduced strain due to lower contact rates, and the establishment of herd immunity could be more rapid and straightforward.

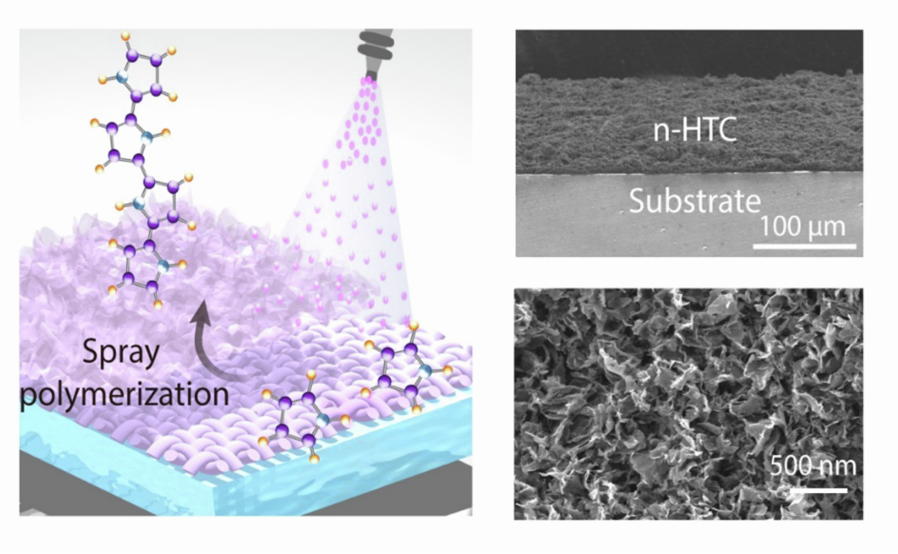

Figure S1. Spray-on polymerizations process and SEM images of as-prepared samples.

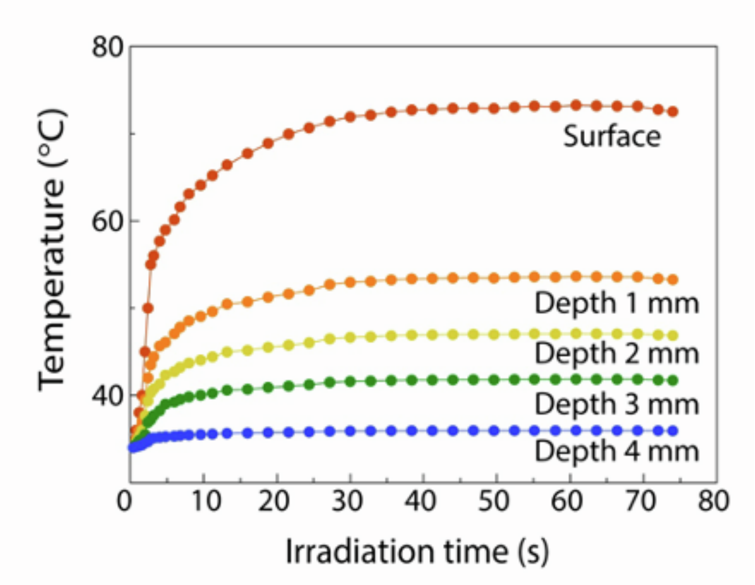

Figure S2. Time-dependent temperature rises at different depths from the sample surface.

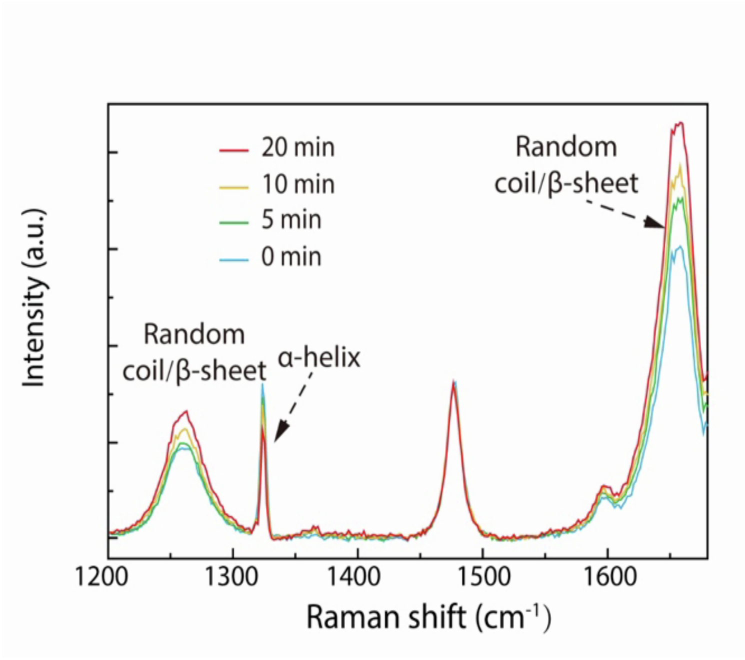

Figure S3. Raman spectra of pathogens measured on n-HTC surface under different contact times. The Raman peaks indicate key proteins secondary structures: a-helix, β-sheet, and random coil, as well as their time-dependent structural transitions.

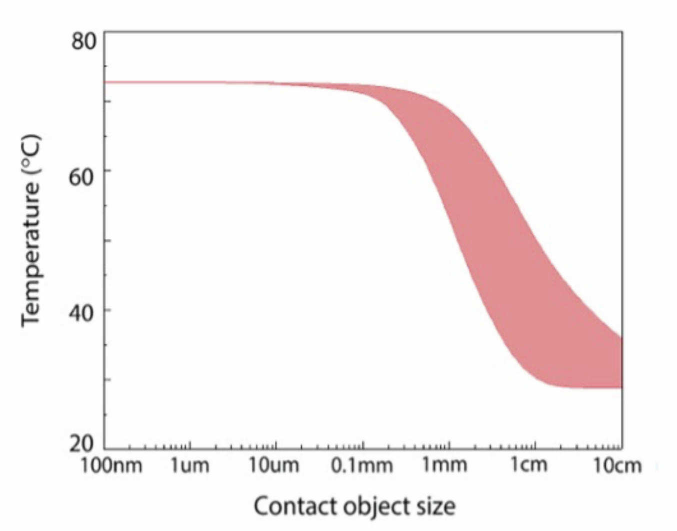

Figure S4. The model simulated object size-dependent temperatures for a smooth surface. The shadowed area accounts for variations in convective heat transfer coefficients in the simulations.
